# The Influence of the Addition of Fruit and Vegetable Concentrates on the Stability of Anthocyanins in Juices from Colored Potatoes

**DOI:** 10.3390/ijms25147584

**Published:** 2024-07-10

**Authors:** Agnieszka Tkaczyńska, Elżbieta Rytel, Alicja Z. Kucharska, Joanna Kolniak-Ostek, Anna Sokół-Łętowska

**Affiliations:** 1 Department of Food Storage and Technology, Wrocław University of Environmental and Life Sciences, 37 Chełmońskiego Str., 51-630 Wrocław, Poland; agnieszka.tkaczynska@upwr.edu.pl; 2 Department of Fruit, Vegetable and Plant Nutraceutical Technology, Wrocław University of Environmental and Life Sciences, 37 Chełmońskiego Str., 51-630 Wrocław, Poland; alicja.kucharska@upwr.edu.pl (A.Z.K.); joanna.kolniak-ostek@upwr.edu.pl (J.K.-O.); anna.sokol-letowska@upwr.edu.pl (A.S.-Ł.)

**Keywords:** potato juices, pigments, concentrates, organic acids, total polyphenols, anthocyanins

## Abstract

One of the factors precluding potato juice application in the food industry is its propensity for enzymatic browning. The addition of fruit and vegetable concentrates rich in organic acids can reduce unfavorable changes in the color of potato juices and influence the content of biologically active compounds. This study aimed to investigate the effect of the addition of natural fruit and vegetable concentrates to potato juices on their color and on the composition and contents of anthocyanin pigments isolated from them. The color, composition and amount of anthocyanins in potato juices and dyes were determined using HPLC-DAD and UHPLC MS/MS liquid chromatography. It was found that the juices without concentrate addition had, on average, 61% lower total polyphenol content and 63% lower anthocyanin content compared with the juices with added concentrates. The isolated pigments contained, on average, 30–40 times more anthocyanins compared with those isolated from the non-purified juices. Furthermore, the addition of concentrates enabled ca. 3–5 times more effective isolation of anthocyanins compared with the samples without these additives. Potato juices and dyes with the addition of concentrates showed a lighter color that did not change over time, compared with samples without concentrates.

## 1. Introduction

Potato juice is a by-product from potato processing and is mainly applied to produce a feedstuff protein concentrate [1,2]. Today, given the high nutritional value of potato protein, particularly including contents of its exogenous amino acids like leucine, lysine, phenylalanine, and threonine, its concentrates are increasingly often used as food additives, enhancing its nutritional value [3]. Apart from high-quality protein, potato juice contains other nutrients and biologically active compounds indigenous to potatoes, in addition to starch and dietary fiber, which are removed from it during starch production [4]. 

In the past, potato juice has been used in folk medicine to treat conditions, e.g., inflammatory conditions of the alimentary tract [1], owing to its high anti-oxidative, anti-inflammatory, and anti-carcinogenic activities. Due to these properties, potato juice may today be considered functional food [1]. This type of food has recently spurred a growing interest among Western populations as a result of their increased health awareness, which has included a change in lifestyle research [5,6].

Juice made from red-fleshed or purple-fleshed potato varieties is a more attractive product compared with that made from light-fleshed potatoes, not only because of its color but also because of its higher content of biologically active substances [7]. The juice produced from colored-flesh potatoes has ca. 2–3-fold higher content of polyphenolic compounds and exhibits 6–7-fold higher antioxidative activity compared with juices made of traditional (light) potato tubers. Phenolic compounds of colored-flesh potatoes and juices made of them primarily include phenolic acids and anthocyanins [8,9,10]. 

One of the factors precluding potato juice application in the food industry is its propensity for enzymatic browning [9]. A change in the color of potato tuber flesh and juices made of it is triggered by the oxidation of chlorogenic and caffeic acids, tyrosine, and other polyphenols by peroxidase and polyphenol oxidase. These processes occurring in light-fleshed potatoes (with yellow and creamy flesh) are well recognized, whereas those observed in potatoes with colored flesh and products made of them follow a different pattern and have not been addressed in the scientific literature [9].

Sulfuric acid and its compounds (sodium acid sulfite, sodium metabisulfite) are used in the food industry to enhance the color of semi-finished products and potato products. Although they exhibit very good antioxidative properties and are inexpensive [11], they present certain drawbacks as their residues may remain in the final products and pose adverse effects on the human body [12]. For this reason, their use raises many controversies among both consumers and food producers; hence, novel and natural additives that may elicit more benefits in the future are searched for and implemented in technological operations [13,14,15,16]. These may include natural organic acids, like citric, oxalic, malic, and tartaric acids, found in fruits, vegetables, and juices made of them [14]. Organic acids of plant raw materials exhibit similar antioxidative properties to sulfur compounds but are harmless to people and animals, and their use does not raise concerns among consumers [13]. Furthermore, their hydroxy acids impart characteristic acidity, and pleasant taste and aroma to fruit–vegetable products. They may also reduce their pH, thereby improving their color stability, particularly in the case of products containing anthocyanin pigments [14]. In addition, organic acids are known for their antioxidative properties, owing to which they may prevent enzymatic browning of fruit or vegetable flesh [15]. This study aimed to investigate the effect of the addition of natural fruit and vegetable concentrates to potato juices on their color and the composition and contents of anthocyanin pigments isolated from them.

## 2. Results and Discussion 

### 2.1. Characterization of Potato Juices

**Figure 1:**
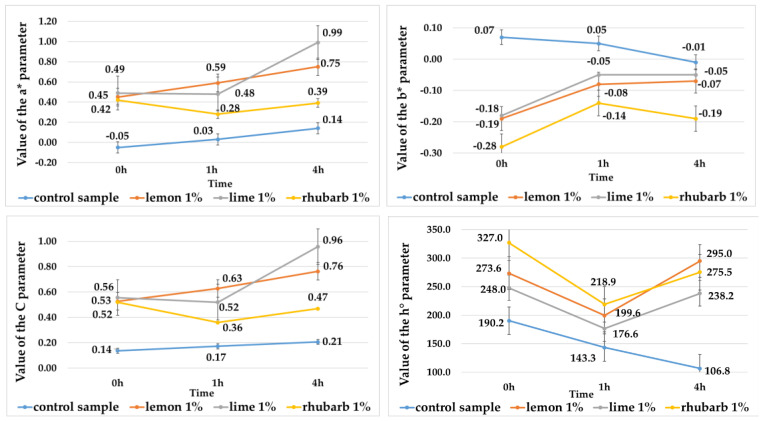
Value of the a*, b*, C, and h° parameters from potato juices of purple-flesh varieties without additives (control sample) and with additives fruit or vegetable concentrates.

**Figure 2:**
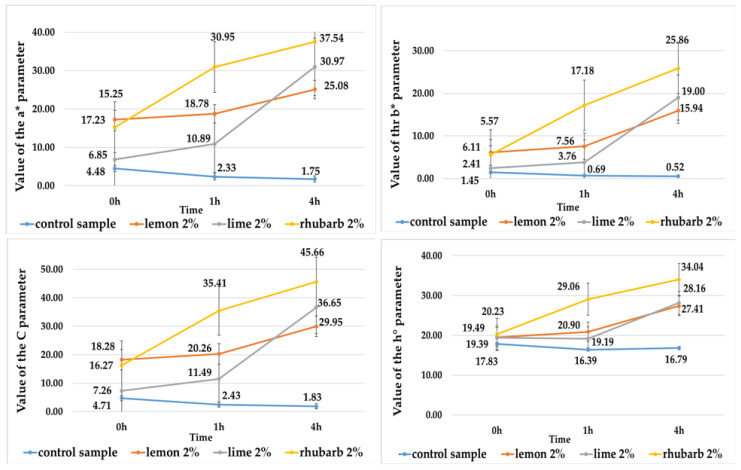
Value of the a*, b*, C, and h° parameters from potato juices of red-flesh varieties without additives (control sample) and with additives fruit or vegetable concentrates.

The juices extracted from red-fleshed and purple-fleshed potato tubers differed significantly in color. Those produced from purple-fleshed potatoes had a darker color (L* = 0.12) (Table 1), as well as a greater contribution of blue (parameter b* = 0.07) (Figure 1) and smaller contribution of red (a = −0.05) (Figure 1) in the color profile, compared with the juices made of the red-fleshed tubers (L* = 0.89) (Table 1), (a* = 4.48) (Figure 2), (b* = 1.54) (Figure 2). Similar findings regarding L*, b* and a* values were also reported by Rytel et al. [17] and Iborra-Bernarda et al. [18] for purple-fleshed potato varieties. The lower L* value of the purple-fleshed potatoes compared with that of the red-fleshed varieties is primarily due to the higher content of anthocyanins and their different compositions [10,19,20]. The juices made from the purple-fleshed potato tubers showed a greater propensity for color darkening. The color of juices was observed to change significantly over time in control samples and red potato juices. Four hours after their preparation, the value of the L* parameter reached L* = 0.02 in juices made of the purple-fleshed tubers (Table 1) and L* = 0.31 in those made of the red-fleshed ones (Table 1). In contrast, Tkaczyńska et al. [9] did not observe any changes in L* values over time in their study on colored-flesh potatoes. The darkening of potato juices may be due to the partial degradation of anthocyanins and their various susceptibilities to oxidation by polyphenol oxidase and peroxidase [9].

The unbeneficial changes in the color of potato juices may be mitigated by reducing their pH, for example [14]. In the present study, this effect was achieved by using concentrates of lemon, lime, and rhubarb juices differing in their contents of organic acids (Table 2). The lemon and lime juice concentrates had the highest contents of citric and malic acids. In turn, the rhubarb juice concentrate had the highest contents of oxalic and citric acids. All analyzed concentrates had very low or trace amounts of ascorbic acid (Table 2). The effects of the addition of concentrated fruit and vegetable juices were observed to differ depending on raw material type. The color of the purple-fleshed potato juices was influenced to the greatest extent by the addition of lemon and lime juice concentrates.

These juices had the highest L* parameter value, ranging from 0.22 to 0.23 (Table 1), and showed greater redness (parameter a* value from 0.45 to 0.49) (Figure 1) and blueness (parameter b* value from −0.18 to −0.19) (Figure 1) compared with the juice without additives (PCS) (L* = 0.12, a* = −0.05, b* = 0.07) (Table 1, Figure 1). When analyzed 4 h post preparation, the color of the purple-fleshed potato juices with the addition of lemon and lime juice concentrates was observed to brighten (its L* value increased), whereas that of the control sample (PCS) was observed to darken (its L* value decreased) (Table 1). Among the juices made of red-fleshed potato tubers, analyzed immediately after preparation, lighter color was measured in those with the addition of concentrates from lemon (L* = 4.13) and rhubarb (L* = 3.52) (Table 1). These juices also showed the greatest increase in the contribution of red (a* = 17.2 and a* = 15.2) (Figure 2) and yellow (b* = 6.11 and b* = 5.57) in the color profile (Figure 2) compared with the RCS sample (L* = 0.89, a* = 4.48, b* = 1.45) (Table 1, Figure 2). After 4 h, the brightest color (parameter L*) (Table 1) and the highest redness (parameter a*) (Figure 2) and yellowness (parameter b*) (Figure 2) were measured in the red-fleshed potato juices with the addition of rhubarb concentrate, compared with the juice without its addition (L* = 0.31, a* = 1.75, b* = 0.52) (Table 1, Figure 2). The juices with the addition of fruit and vegetable concentrates also had higher color chroma values, which in the case of purple-fleshed potato juices ranged from C = 0.52 (rhubarb juice concentrate addition) to C = 0.56 (lime juice concentrate addition) (Figure 1) on average, compared with PCS (C = 0.14) (Figure 1). Their color hue angle was also higher, ranging from h° = 248.04 (lime juice concentrate addition) to h° = 326.0 (rhubarb juice concentrate addition) (Figure 1) on average, compared with PCS (h° = 190.25) (Figure 1). After 4 h of juice preparation, the C and h° values were observed to decrease in both the juices with concentrate addition and PCS (Figure 1). In the case of juices extracted from the red-fleshed potato varieties and analyzed immediately after preparation, the C value increased from 7.26 (lime juice concentrate addition) to 18.28 (lemon juice concentrate addition) (Figure 2) and the h° value increased from h° = 19.39 (lime juice concentrate addition) to h° = 20.23 (rhubarb juice concentrate addition) (Figure 2), compared with RCS (C = 4.71, h° = 17.83). Then, 4 h after production, the values of these color parameters increased in the potato juices with the addition of fruit and vegetable juice concentrates, and decreased over time in the RCS sample (Figure 2). According to Kościuk et al. [21], citric acid found in citrus fruits mitigates enzymatic browning. The concentrates used in the present study had a high concentration of this acid (ranging from 217.2 mg/mL to 370.7 mg/mL) compared with the other analyzed organic acids (Table 2). Our previous study, Rytel et al. [17], demonstrated the stability of anthocyanins to be affected by temperature and pH. The pH of the potato juices without the addition of fruit and vegetable concentrates ranged from 6.0 to 6.3 (Table S4), whereas after concentrate addition, their pH increased from 3.2 to 4.7 (Table S4). Many authors [14,17,22,23,24] have confirmed that pH reduction in plant raw materials via their acidification inhibits their enzymatic browning, thereby enhancing their color and improving their stability. Those authors attributed the observed anthocyanin content decay to possible enzymatic reactions. 

Based on the research conducted, it was found that the addition of fruit and vegetable juice concentrates also affected the contents of total polyphenols (TPs) and anthocyanins in the potato juices. The juices without concentrate addition (PCS, RCS) had 61% lower TP content and 63% lower anthocyanin content on average compared with the juices with added concentrates (Table 3). The highest contents of total polyphenols and anthocyanins were determined in the juices extracted from purple-fleshed potatoes with the addition of lemon concentrate (Table 3). The higher contents of TP and anthocyanins in the potato juices with the addition of fruit and vegetable concentrates may be attributed to the effects of organic acids that occur naturally in most fruits and vegetables. The change to a more acidic pH contributes to the stabilization of polyphenolic compounds and prevents their degradation. According to Sun et al. [25], the pH of the environment affects the total polyphenol content, as they demonstrated a higher content of these compounds in sweet potatoes at a pH ranging from 5.0 to 7.0. Such an effect was, however, not confirmed in our previous study [17]. In turn, most authors [13,14,15] have confirmed the positive impact of an acidic environment on the composition and contents of anthocyanins. A low-pH environment affects the intensity of the color of anthocyanins and improves their stability. Natural organic acids contained in fruit and vegetable concentrates protect the color of potato juices by preventing enzymatic darkening processes, changes occurring in the air, and the action of enzymes.

The potato juices analyzed in this study differed significantly in this respect (Table 4 and Table 5). Anthocyanins identified in the purple-fleshed potato juices included malvidin and petunidin, whereas those identified in the red-flesh potato juices were pelargonidin with derivatives and cyanidin (Table 4 and Table 5). Other authors [8,17] have also demonstrated petunidin to be the major anthocyanin of purpled-fleshed potato tubers and pelargonidin to be the major anthocyanin of red-fleshed ones. However, Ngcobo et al. [16] identified cyanidin and peonidin in purple sweet potatoes. The addition of fruit and vegetable concentrates affected the contents of individual anthocyanins in juices made of colored-flesh potatoes (Table 4 and Table 5). A positive effect of the addition of lime and rhubarb concentrate on the stability of anthocyanins in juices from purple potato varieties and lime and lemon in juices from red potato varieties was demonstrated (Table 4 and Table 5).

Many authors [13,14,15,16,17,22,23,24,25] have confirmed that the structure and stability of anthocyanins in fruits and vegetables depend on raw material type and environment pH. Fan et al. [26] demonstrated that anthocyanins found in purple-fleshed potatoes were degraded along with a pH increase above 5.0, while they remained stable at pHs of 2.0–5.0. Li et al. [24] also reported a positive influence of acidic pH on the stability of anthocyanin structure and color in purple-fleshed potato varieties. At a low pH (from 1.0 do 3.0), anthocyanins are believed to occur in the form of a red flavylium cation, which is their most stable form [26]. With a pH increase (from 3.0 to 6.0), their color turns lighter and they transform into colorless carbinol pseudo-bases, yellow chalcones, and purple quinoidal bases. In turn, blue anionic quinoidal bases prevail at pH 6.0–7.0, whereas alkaline environments cause anthocyanin degradation [14,15,26,27].

### 2.2. Characterization of Anthocyanin Pigments

**Figure 3:**
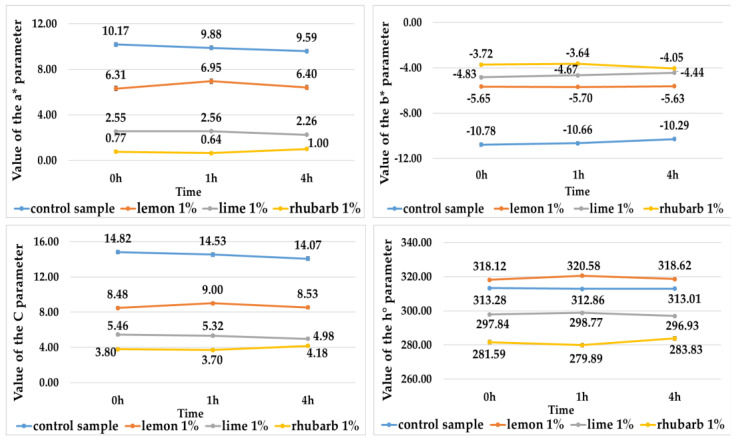
Value of the a*, b*, C, and h° parameters, i.e., pigments from potato juices of purple-flesh varieties without additives (control sample) and with additives, i.e., fruit or vegetable concentrates.

**Figure 4:**
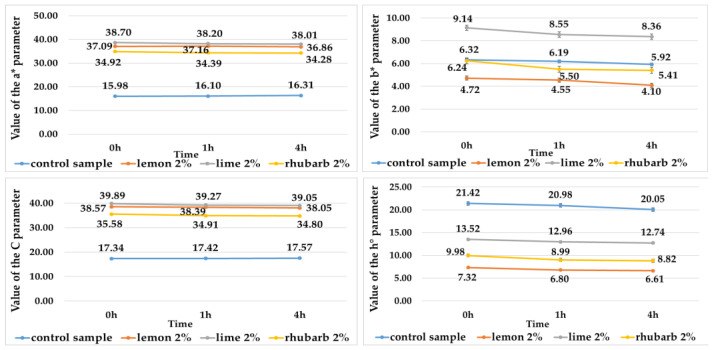
Value of the a*, b*, C, and h° parameters, i.e., pigments from potato juices of red-flesh varieties without additives (control sample) and with additives, i.e., fruit or vegetable concentrates.

The addition of fruit and vegetable concentrates to potato juices influenced the color of anthocyanin pigments isolated from them (Table 6, Figure 3 and Figure 4). 

All isolated pigments had a lighter color (indicated by a higher L* value) compared with the potato juices (Table 1 and Table 6). The lightest color was found for the pigments isolated from red-fleshed potato juices (L* from 18.6 to 22.4) (Table 6). Anthocyanin pigments obtained from juices without and with the addition of fruit and vegetable concentrates did not darken over time, and their color was stable (Table 6, Figure 3 and Figure 4). Once isolated from the purple-fleshed potato juices, their color was affected to the largest extent by the addition of lime juice concentrate, whereas the color of pigments isolated from the red-fleshed potato juices was affected to the largest extent by the addition of lemon concentrate (Table 6). The anthocyanin pigments isolated from the red-fleshed potato juices had a greater contribution of red and a smaller contribution of yellow in the color profile, compared with the potato juices (Figure 2 and Figure 4). In turn, the pigments isolated from the purple-fleshed potato juices had a greater contribution of red and blue, compared with the potato juices (Figure 1 and Figure 3). In addition, the anthocyanin pigments showed higher values and smaller changes in the C and h° color parameters over time, compared with the potato juices (Figure 1, Figure 2, Figure 3 and Figure 4).

The addition of fruit and vegetable juice concentrates also positively influenced the content and composition of anthocyanins in the juices after their purification (pigments) (Table 7 and Table 8).

The isolated pigments had a ca. 30–40-fold higher content of anthocyanins on average than the non-purified juices. In addition, the reduction in the potato juices’ pH caused by the addition of natural fruit and vegetable concentrates contributed to even more effective isolation of anthocyanins as the purified juices with concentrates had 3–5 times more anthocyanins compared with the control juices (Table 7 and Table 8). Fruit and vegetable concentrates contain organic acids, which may form permanent complexes with anthocyanins. The stable structure of the latter affects not only their composition but also their color stability. This study also showed that the composition and content of anthocyanins isolated from purple-fleshed potatoes was affected to a greater extent by the addition of lemon and rhubarb concentrates, whereas those of anthocyanins isolated from red-fleshed potato juices was affected to a greater extent by the addition of lime and rhubarb concentrates (Table 7 and Table 8). 

The analysis of anthocyanin stability provides important information in terms of their applicability as food additives [28]. Many authors [9,13,16,17,19,24,28] have reported that anthocyanins are non-stable compounds that may undergo irreversible (permanent) and reversible changes in the aquatic environment, which affect their color. Their stability depends, most of all, on their structure, environment (pH, contents of sugars and their degradation products, content of enzymes, and presence of co-pigments), and external conditions (light, temperature, access to oxygen). Molecules of anthocyanins found in potatoes and potato juices contain acylated groups, which have a positive impact on the stability of these compounds [9,13,14]. Furthermore, anthocyanins are capable of forming complexes with organic acids, alkaloids, flavonoids, amino acids, nucleotides, polysaccharides, and metals, for example, among others [13]. Both the color and structure of co-pigments formed with anthocyanins are very stable. 

## 3. Materials and Methods

### 3.1. Colored Potato Juices

The experimental material included potato juices and anthocyanin pigments isolated from these juices. Potato juices were made from tubers of one purple-fleshed potato variety, Violet Queen, and from tubers of two red-fleshed varieties: Magenta Love and Mulberry Beauty. Potatoes were sourced directly from producers, from the vegetative seasons of 2020 and 2021. Lemon, lime, and rhubarb juice concentrates (Döhler Holland B.V., Oosterhout, The Netherlands) were also used in the study. 

#### 3.1.1. Preparation of Potato Juices 

Ca. 30 kg of potatoes were randomly selected from producers’ batches, washed, and dried with a towel. Juice was pressed from non-peeled tubers using a Robot Coupe J100 automatic juice extractor. Aqueous solutions of lemon, lime, and rhubarb juice concentrate were directly added to potato juices during their production, in the following concentrations: 1% in the case of juices made of purple-fleshed tubers and 2% in the case of those made of red-fleshed tubers. The addition of fruit and vegetable juice concentrates was calculated after taking into account their dry weight. The concentrations of the fruit and vegetable juice concentrates were adjusted empirically based on color measurements of potato juices with their addition (Table S1). 

The juices with added concentrates were left to stand in a dark place for 45 min to allow the starch to separate via sedimentation. Afterward, they were filtered through a filtration cloth and centrifuged using an MPW-351R centrifuge at 1000 rpm and a temperature of 9 °C for 10 min to obtain clear juice. Juice without concentrate addition served as the control sample.

#### 3.1.2. Preparation of Potato Pigments

Anthocyanin pigments were isolated from potato juices with and without the addition of fruit and vegetable juice concentrates using gel chromatography. A chromatographic column was filled with “Amberlite XAD 16” resin, potato juice was directly injected into the column, and anthocyanin pigments were eluted from the resin using 70% ethanol. Next, the pigments were concentrated via ethanol evaporation in a vacuum evaporator (bath temp. 40 °C, 239 mbar). The concentrated pigments were transferred onto Petri dishes and dried at room temperature under a fume hood for 24 h. The resulting powder was frozen at temp. −18 °C for further analyses. 

Samples of potatoes (ca. 1 kg) and potato juices (ca. 1 L) were lyophilized in a Christ Alpha 1-4 LSCplus freeze-dryer (Osterode am Hatz, German) at the following parameters: a pressure of 63 Pa, shelf heating temperature of 30 °C, and duration from 24 h (potatoes) to 48 h (potato juices). The lyophilized samples were stored at a temperature of −18 °C in closed containers until analysis.

### 3.2. Analytical Methods

The juices with fruit and vegetable concentrate addition and the control sample (PCS, RCS) (juice without concentrates) were subjected to color analysis with the colorimetric method (Table S2) [29,30]. The content of total polyphenols (TPs) of the lyophilized samples was determined [10,31], and for the content and composition of anthocyanins (TA), the following liquid chromatography methods were used: HPLC-DAD and UHPLC MS/MS [32]. The fruit and vegetable concentrates were analyzed for the contents of organic acids, oxalic, malic, lactic, citric, and ascorbic, with the HPLC method [33]. 

In turn, the pigments isolated from potato juices were subjected to color analysis with the calorimetric method (Table 6 and Table S3) [29,30] and to the determination of the content and composition of anthocyanins (TA) with the following liquid chromatography methods: HPLC-DAD and UHPLC MS/MS [32].

#### 3.2.1. Color Analysis with the Konica Minolta CR-5 Camera according to the Hunter Scale (Lab)

The color analysis of the juices and pigments was performed with a Konica Minolta CR-200 measuring apparatus calibrated to the Hunter scale’s L, a, and b units. Color measurements were conducted immediately after the preparation of the juices with and without added concentrates as well as 1 h and 4 h after their production [29].

Color space parameters, hue angle (h°) and chroma (C), were computed based on a* and b* values:

- Hue angle = Arctan (b*/a*)
- Chroma = ((a*^2^) + (b*^2^)) ^0.5^ [30].

#### 3.2.2. Extraction of Polyphenols and Anthocyanins

The lyophilized samples of potatoes and juices were subjected to the extraction with a 70% aqueous acetone solution acidified with 0.1% acetic acid. Two-gram samples of the lyophilizates were collected for analysis. The mixture was mixed with a Vortex stirrer, then placed in a SONIC-9 ultrasonic water bath for 5 min (21 °C/ 5 min/40 KHz/2 × 160 W), and centrifuged using an MPW-351R centrifuge (5 min/10,000 rp m/temp. 4 °C). The extraction was repeated two more times. Afterwards, the acetone–water layer was separated using chloroform to remove lipophilic compounds. The color acetone–water fraction was collected and evaporated on a Büchi rotary evaporator (Merck, Darmstadt, Germany) until the acetone was completely removed. The remaining extract was brought to a volume of 5 mL using 50% methanol. The samples were stored in a freezer at −20 °C until analysis. Before we conducted chromatographic analyses, the samples were filtered through “Nylon 6” 0.22 µm filters [31].

#### 3.2.3. Total Polyphenols Content

The total polyphenol content (TP) was determined with the Folin–Ciocalteau colorimetric method [34]. Determinations were performed using 0.1 mL samples of extracts (prepared as in Section 3.2.2), completed with 2 mL of distilled water and 0.2 mL of the Folin–Ciocalteau reagent. Next, 1 mL of a 20% aqueous sodium carbonate solution was added to the mixture. After one hour, absorbance was measured at a wavelength of 765 nm with the spectrophotometric method. The results were presented as mg of gallic acid (GAE/1 g expressed per dry weight of the sample) [10].

#### 3.2.4. Quantification of Anthocyanins by HPLC-PDA

The content of anthocyanins (TAs) was determined in accordance with Kucharska et al. [32] using a Dionex (Waltham, MA, USA) HPLC system equipped with an Ultimate 3000 model of a diode array detector, an LPG-3400A quaternary pump, an EWPS-3000SI autosampler and a TCC-3000SD thermostated column compartment, controlled by Chromeleon v.6.8. software. The Cadenza Imtakt column C5-C18 (75 × 4.6 mm, 5 µm; Portland, OR, USA) was used for HPLC. The following solvents constituted the mobile phase: 45% formic acid (solvent A) and 100% acetonitrile (solvent B). The following elution conditions were applied: 0–1 min 5% B in A; 1–20 min 25% B in A; 20–27 min 100% B in A; and 27–30 min 5% B in A. The flow rate was 1 mL/min, and the injection volume was 40 µL. The column was operated at 30 °C. Anthocyanins were monitored at 520 nm and their content was expressed in cyanidin 3-*O*-glucoside equivalents (CygE)/100 g dry mass (dm).

#### 3.2.5. Determination of the Content and Composition of Anthocyanins via UHPLC MS/MS Liquid Chromatography

The compounds were identified using the Acquity liquid chromatography system (UPLC) coupled with quadrupole time-of-flight (Q-TOF) MS (UPLC/Synapt Q-TOF MS, Waters Corp., Milford, MA, USA), with an ionization source provided by electrospraying (ESI). The separation was performed on an Acquity BEH C18 column (100 mm × 2.1 mm id, 1.7 µm; Waters), with a mixture (*v*/*v*) of 2.0% formic acid (A) and acetonitrile (B) as the mobile phase. The gradient program was as follows: initial conditions—1% B in A; 12 min—25% B in A; 12.5 min—100% B; and 13.5 min—1% B in A. The flow rate was 0.45 mL/min, and the sample injection volume was 5 μL. The column operated at a temperature of 30 °C. UV-Vis absorption spectra were registered online during UPLC analysis, and spectral measurements were performed in the wavelength range of 200–600 nm, in 2 nm ramps. The main parameters of Q-TOF MS work were as follows: capillary voltage: 2.0 kV; cone voltage: 40 V; gas flow rate on the cone: 11 L/h; collision energy: 28–30 eV; source temperature: 100 °C; desolvation temperature: 250 °C; collision gas: argon; desolvation gas (nitrogen); flow rate: 600 L/h; data acquisition range: m/z 100–2000 Da; ionization mode: negative and positive. Data were collected using Mass-LynxTM V 4.1 software. The content of anthocyanins was monitored at the wavelength of ƛ = 520 nm [32].

#### 3.2.6. Determination of the Content of Organic Acids Using High-Performance Liquid Chromatography (HPLC)

Organic acids were quantified with the HPLC metod using Prominence-i LC-2030C Plus, made by Shimadzu Corporation (Kyoto, Japan), equipped with LC-2030 UV detector, Supelcosil LC-18 (25 cm × 4.6 mm, 4 μm) analytical column, (Supelco Inc., Bellefonte, PA, USA), at a temperature of 15 °C and a liquid phase (0.001 N sulfuric acid) flow rate of 0.7 mL min^−1^. Oxalic, malic, lactic, and citric acids were detected at a wavelength of 210 nm, whereas ascorbic acid was detected at a wavelength of 254 nm, and identified based on chromatograms of pure chemical standards [33].

#### 3.2.7. Statistical Analysis

The results were processed using one-way and two-way analysis of variance using Statistica 13.1 package, with the least significant difference (LSD) and homogenous groups determined using the Duncan test at a significance level of α = 0.05. 

Determinations of the contents and composition of anthocyanins and organic acids were conducted in two laboratory replications, whereas determinations of polyphenol content and color analyses were conducted in six laboratory replications. The results reported in the manuscript represent mean values from the laboratory replications and two study years (growing seasons).

## 4. Conclusions

The juices made from the purple-fleshed potatoes had a darker color (L* = 0.12), a greater contribution of blue (b* = 0.07), and a lower contribution of red (a = −0.05) in the color profile, compared with those made from the red-fleshed potatoes (L* = 0.89, a* = 4.48, b* = 1.54). Over time, the color of potato juices turned darker, and the value of the L* parameter decreased to L* = 0.02 for the purple-fleshed potato juices and to L* = 0.31 for the red-fleshed potato juices. The addition of fruit and vegetable concentrates had a positive effect on the color of potato juices, which was lighter, had a higher chroma value, and did not change over time. The color of the purple-fleshed potato juices was affected to the greatest extent by the addition of lemon and lime concentrates, whereas that of the red-fleshed potato juices was affected to the greatest extent by lemon and rhubarb concentrates. The addition of fruit and vegetable juice concentrates also affected the contents of total polyphenols and anthocyanins in the potato juices. The juices without concentrate addition had 61% lower total polyphenol content and 63% lower anthocyanin content on average compared with the juices with added concentrates.

Purification of potato juices contributed to the preservation of a higher amount of anthocyanins. The isolated pigments contained ca. 30–40 times more anthocyanins on average compared with those isolated from the non-purified juices. Furthermore, the addition of fruit and vegetable concentrates enabled the ca. 3–5-fold more effective isolation of anthocyanins compared with the samples without these additives. It was also demonstrated that the composition and contents of anthocyanins isolated from the purple-fleshed potatoes were affected to a greater extent by the addition of lemon and rhubarb concentrates, whereas those of anthocyanins found in the red-fleshed potatoes— were affected to a greater extent by the addition of lime and rhubarb concentrates.

Conducting research on the use of potato juice on a larger scale, e.g., for the production of dyes, is advisable not only because of the management of burdensome production waste, but above all as a new and cheap source of biologically active compounds.

Anthocyanin-rich extracts from red- and purple-fleshed potato juices have high potential as natural colorants, with multiple applications in the food industry. 

## Figures and Tables

**Table 1:** Value of the L* parameter from potato juices of red- and purple-flesh varieties: without additives (control sample) and with additives fruit or vegetable concentrates.

		0 h	1 h	4 h	
Flesh Color	Variety	L*	L*	L*	LSD
purple	control sample	0.12 ± 0.07 ^aB^	0.10 ± 0.06 ^aB^	0.02 ± 0.06 ^aA^	0.06
	lemon 1%	0.22 ± 0.07 ^bA^	0.23 ± 0.07 ^bA^	0.29 ± 0.06 ^bA^	0.11
	lime 1%	0.23 ± 0.10 ^bA^	0.20 ± 0.09 ^bA^	0.29 ± 0.06 ^bA^	0.10
	rhubarb 1%	0.17± 0.13 ^abA^	0.16 ± 0.07 ^abA^	0.24 ± 0.06 ^bA^	0.90
	LSD	0.09	0.07	0.11	
red	control sample	0.89 ± 0.40 ^aB^	0.40 ± 0.20 ^aA^	0.31 ± 0.23^aA^	0.24
	lemon 2%	4.13 ± 1.70 ^bA^	4.48 ± 2.28 ^bA^	9.51 ± 3.29^cB^	3.74
	lime 2%	1.42 ± 0.16 ^aA^	2.24 ± 0.58 ^aA^	11.21 ± 3.17^bB^	3.44
	rhubarb 2%	3.52 ± 1.92 ^bA^	10.52 ± 0.32 ^bB^	16.14 ± 4.00^dC^	2.12
	LSD	1.07	1.07	0.98	

Data are expressed as the mean and standard deviation (± SD), n = 12. Results in the same column followed by different letters indicate significant differences according to Duncan’s test at *p* < 0.05 between different flesh colors and varieties (small letters), and between time (big letters), as determined and via one-way ANOVA. LSD—least significant difference.

**Table 2:** Content of organic acids (mg/100 g d. m) in fruit and vegetable concentrates.

	Concentrate		
Acid	Lemon	Lime	Rhubarb
Oxalic	0.96 ^a^	1.00 ^a^	345.2 ^b^
Malic	55.1 ^b^	42.1 ^a^	73.3 ^c^
Lactic	6.70 ^a^	8.50 ^c^	7.58 ^b^
Citric	340.2 ^b^	370.7 ^c^	217.2 ^a^
Ascorbic	1.83 ^b^	0.02 ^a^	0.02 ^a^

Data are expressed as the mean, n = 6. Results in the same column followed by different letters indicate significant differences according to Duncan’s test at *p* < 0.05 between the additions of concentrates as determined via one-way ANOVA.

**Table 3:** Content of total polyphenols (mg GAE/1 g d. m) and anthocyanins (mg/100 g d. m) in potato juices without additives (control sample) and with additives fruit or vegetable concentrates.

Concentrate	Variety of Potato Juice	Polyphenol Content	Anthocyanin Content
control sample	Violet Queen	5.65 ± 0.15 ^a^	61.0 ± 1.91 ^ab^
lemon 10%	Violet Queen	26.0 ± 1.54 ^c^	288.9 ± 8.54 ^d^
lime 10%	Violet Queen	24.8 ± 1.60 ^c^	148.4 ± 8.05 ^bc^
rhubarb 10%	Violet Queen	16.6 ± 0.56 ^b^	61.6 ± 1.05 ^ab^
control sample	Mulberry Beauty	6.07 ± 0.53 ^a^	24.9 ± 0.69 ^a^
lemon 20%	Mulberry Beauty	25.4 ± 1.97 ^c^	128.8 ± 1.09 ^bc^
lime 20%	Mulberry Beauty	20.7 ± 1.57 ^bc^	185.4 ± 10.40 ^c^
rhubarb 20%	Mulberry Beauty	19.7 ± 1.59 ^bc^	188.0 ± 11.77 ^c^
control sample	Magenta Love	4.27 ± 0.70 ^a^	23.9 ± 1.93 ^a^
lemon 20%	Magenta Love	22.1 ± 1.85 ^bc^	159.4 ± 10.41 ^bc^
lime 20%	Magenta Love	20.6 ± 1.88 ^bc^	142.4 ± 9.11 ^bc^
rhubarb 20%	Magenta Love	20.0 ± 0.70 ^bc^	132.9 ± 9.47 ^bc^
	LSD	6.84	89.81

Data are expressed as the mean and standard deviation (± SD), n = 12; n = 6. Results in the same column followed by different letters indicate significant differences according to Duncan’s test at *p* < 0.05 between a variety of potato juices, as determined by two-way ANOVA. LSD—least significant difference.

**Table 4:** Content of identified anthocyanins (mg/100 g d. m) in purple-flesh potato juice without additives (control sample) and with additives fruit or vegetable concentrates.

Compound	Violet Queen			
	Control Sample	Lemon 2%	Lime 2%	Rhubarb 2%
Petunidin 3-caffeoylrutinoside 5-glucoside	1.69 ^a^	5.78 ^c^	3.27 ^b^	1.70 ^a^
Petunidin 3-coumaroylrutinoside 5-glucoside	40.60 ^b^	210.8 ^d^	92.36 ^c^	27.08 ^a^
Petunidin 3-feruloylrutinoside 5-glucoside	1.00 ^a^	4.36 ^c^	2.20 ^b^	0.82 ^a^
Malvidin 3-coumaroylrutinoside 5-glucoside	16.84 ^a^	65.42 ^d^	48.64 ^c^	31.13 ^b^
Malvidin 3-feruloylrutinoside 5-glucoside	0.84 ^a^	2.59 ^c^	1.90 ^b^	1.26 ^b^

Results in the same column followed by different letters indicate significant differences according to Duncan’s test at *p* < 0.05 between varieties of potato juices (for each variant separately), as determined via two-way ANOVA.

**Table 5:** Contents of identified anthocyanins (mg/100g d. m) in red-flesh potato juice without additives (control sample) and with additives fruit or vegetable concentrates.

Compound	Mulberry Beauty				Magenta Love			
	Control Sample	Lemon 2%	Lime 2%	Rhubarb 2%	Control Sample	Lemon 2%	Lime 2%	Rhubarb 2%
Pelargonidin 3-rutinoside 5-glucoside	1.18 ^a^	13.33 ^b^	13.97 ^b^	13.66 ^b^	1.42 ^a^	7.89 ^c^	7.26 ^c^	6.69 ^b^
Pelargonidin derivative isomer 1	0.56 ^a^	2.77 ^b^	3.89 ^c^	3.81 ^c^	0.58 ^a^	1.46 ^c^	1.51 ^c^	1.21 ^b^
Cyanidin derivative	-	-	-	-	0.60 ^a^	1.16 ^b^	1.39 ^c^	1.11 ^b^
Pelargonidin derivative isomer 2	0.64 ^a^	2.64 ^b^	2.85 ^c^	2.77 ^b^	-	7.23	5.05	5.81
Pelargonidin 3-coumaroylrutinoside 5-glucoside isomer 1	0.54	-	-	-	0.56	-	-	-
Pelargonidin 3-caffeoylrutinoside 5-glucoside isomer 1	0.92 ^a^	2.65 ^b^	4.05 ^cd^	4.70 ^d^	1.40 ^a^	5.76 ^c^	4.81 ^b^	4.55 ^b^
Pelargonidin 3-caffeoylrutinoside 5-glucoside isomer 2	0.86 ^a^	3.03 ^b^	3.53 ^c^	3.60 ^c^	1.05	-	-	-
Pelargonidin derivative isomer 3	-	-	2.59	-	-	-	-	-
Pelargonidin 3-coumaroylrutinoside 5-glucoside isomer 2	2.03 ^a^	4.97 ^b^	6.67 ^c^	6.60 ^c^	1.34 ^a^	3.04 ^c^	2.23 ^b^	2.55 ^b^
Pelargonidin 3-coumaroylrutinoside 5-glucoside isomer 3	17.23 ^a^	93.80 ^b^	140.8 ^c^	143.6 ^c^	16.07 ^a^	125.4 ^d^	114.5 ^c^	104.7 ^b^
Pelargonidin 3-feruloylrutinoside 5-glucoside	1.02 ^a^	4.98 ^b^	7.06 ^c^	7.64 ^c^	1.09	7.47	6.40	6.28
Cyanidin derivative	-	-	-	-	0.30	-	-	-
Pelargonidin 3-coumaroylrutinoside 5-glucoside isomer 4	0.50	-	-	-	0.74	-	-	-
Pelargonidin 3-coumaroylrutinoside 5-glucoside isomer 5	0.28 ^a^	1.18 ^b^	2.53 ^c^	3.12 ^d^	-	-	-	-

Results in the same column followed by different letters indicate significant differences according to Duncan’s test at *p* < 0.05 between varieties of potato juices (for each variant separately), as determined via two-way ANOVA.

**Table 6:** Value of the L* parameter pigments from potato juices of red- and purple-flesh varieties without additives (control sample) and with additives, i.e., fruit or vegetable concentrates.

		0 h	1 h	4 h	
Flesh Color	Variety	L*	L*	L*	LSD
purple	control sample	8.36 ± 0.08 ^aA^	8.28 ± 0.49 ^aA^	8.93 ± 0.28 ^aB^	0.40
	lemon 1%	11.56 ± 0.47 ^bA^	11.58 ± 1.53 ^bA^	13.80 ± 0.59 ^cB^	1.21
	lime 1%	13.05 ± 0.52 ^cA^	14.67 ± 0.50 ^cB^	14.79 ± 0.14 ^dB^	0.52
	rhubarb 1%	11.78 ± 0.55 ^bB^	11.59 ± 0.59 ^bB^	10.86 ± 0.45 ^bA^	0.65
	LSD	0.51	1.07	0.48	
red	control sample	18.57 ± 3.5 ^aA^	19.06 ± 3.2 ^aA^	18.96 ± 3.7 ^aA^	1.34
	lemon 2%	22.44 ± 4.17 ^aA^	22.11 ± 3.5 ^aA^	22.29 ± 4.33 ^aA^	3.58
	lime 2%	19.50 ± 3.84 ^aA^	19.93 ± 3.70 ^aA^	20.14 ± 4.73 ^aA^	3.09
	rhubarb 2%	18.78 ± 2.53 ^aA^	19.16 ± 4.00 ^aA^	19.64 ± 3.01 ^aA^	4.29
	LSD	7.43	7.27	7.41	

Data are expressed as the mean and standard deviation (± SD) n = 12. Results in the same column followed by different letters indicate significant differences according to Duncan’s test at *p* < 0.05 05 between different flesh colors and varieties (small letters), and between time (big letters) as determined and by one-way ANOVA. LSD—last significant difference.

**Table 7:** Content of identified anthocyanins (mg/100 g d. m) in purple-flesh pigments from potato juice without additives (control sample) and with additives, i.e., fruit or vegetable concentrates.

Compound	Violet Queen			
	Control Sample	Lemon 2%	Lime 2%	Rhubarb 2%
Petunidin 3-caffeoylrutinoside 5-glucoside	50.76 ^a^	291.3 ^c^	273.3 ^b^	288.3 ^bc^
Petunidin 3-coumaroylrutinoside 5-glucoside	1209.9 ^c^	1432.7 ^d^	713.2 ^b^	1151.6 ^b^
Petunidin 3-feruloylrutinoside 5-glucoside	49.40 ^a^	537.8 ^d^	461.5 ^b^	487.7 ^c^
Malvidin 3-coumaroylrutinoside 5-glucoside	317.36 ^a^	1048.8 ^b^	1128.0 ^c^	1123.0 ^c^
Malvidin 3-feruloylrutinoside 5-glucoside	32.01 ^a^	299.1 ^b^	300.5 ^b^	297.5 ^b^
Total anthocyanins (TA)	1659.5 ^a^	3609.7 ^d^	2876.5 ^b^	3348.1 ^c^

Results in the same column followed by different letters indicate significant differences according to Duncan’s test at *p* < 0.05 between varieties of pigments, as determined via two-way ANOVA.

**Table 8:** Content of identified anthocyanins (mg/100 g d. m) in red-flesh pigments from potato juice without additives (control sample) and with additives, i.e., fruit or vegetable concentrates.

Compound	Mulberry Beauty				Magenta Love			
	Control Sample	Lemon 2%	Lime 2%	Rhubarb 2%	Control Sample	Lemon 2%	Lime 2%	Rhubarb 2%
Pelargonidin 3-rutinoside 5-glucoside	78.89 ^a^	553.8 ^b^	552.6 ^b^	640.5 ^c^	28.57 ^a^	431.9 ^b^	438.6 ^bc^	443.6 ^c^
Pelargonidin derivative isomer 1	40.44 ^a^	298.1 ^bc^	292.5 ^b^	306.1 ^c^	-	278.6	278.8	277.5
Pelargonidin derivative isomer 2	40.43 ^a^	285.5 ^b^	282.2 ^b^	297.1 ^c^	-	-	-	-
Cyanidin derivative	-	-	-	-	18.82 ^a^	442.3 ^b^	437.8 ^b^	440.1 ^b^
Pelargonidin 3-coumaroylrutinoside 5-glucoside izomer 1	34.10	-	-	-	-	-	-	-
Pelargonidin 3-caffeoylrutinoside 5-glucoside isomer 1	35.56 ^a^	294.6 ^bc^	285.2 ^b^	303.0 ^c^	17.84 ^a^	338.5 ^d^	315.0 ^b^	329.7 ^c^
Pelargonidin 3-caffeoylrutinoside 5-glucoside isomer 2	32.96 ^a^	314.8 ^b^	318.0 ^bc^	331.1 ^c^	-	350.6	339.9	345.1
Pelargonidin 3-coumaroylrutinoside 5-glucoside izomer 2	85.70 ^a^	318.5 ^bc^	315.5 ^b^	333.6 ^c^	19.70 ^a^	285.9 ^c^	278.8 ^b^	288.4 ^c^
Pelargonidin 3-coumaroylrutinoside 5-glucoside izomer 3	487.89 ^a^	2130.9 ^b^	2186.6 ^c^	2577.7 ^d^	31.65 ^a^	2606.4 ^d^	2304.0 ^b^	2507.2 ^c^
Pelargonidin 3-feruloylrutinoside 5-glucoside	31.34 ^a^	332.8 ^b^	333.2 ^b^	362.8 ^c^	17.40 ^a^	356.4 ^bc^	345.4 ^b^	364.2 ^c^
Pelargonidin 3-coumaroylrutinoside 5-glucoside izomer 4	45.67	-	-	-	-	-	-	-
Pelargonidin 3-coumaroylrutinoside 5-glucoside isomer 5	19.15 ^a^	447.4 ^b^	447.2 ^b^	443.6 ^b^	-	-	-	-
Total anthocyanins (TA)	932.1 ^a^	4978.1 ^b^	5012.8 ^c^	5595.4 ^d^	134.0 ^a^	5090.4 ^d^	4737.8 ^b^	4995.8 ^c^

Results in the same column followed by different letters indicate significant differences according to Duncan’s test at *p* < 0.05 between varieties of pigments, as determined via two-way ANOVA.

## Data Availability

Data will be made available upon request.

## Supplementary Materials

The following supporting information can be downloaded at https://www.mdpi.com/article/10.3390/ijms25147584/s1.
