# Supplementary material for: The Influence of the Addition of Fruit and Vegetable Concentrates on the Stability of Anthocyanins in Juices from Colored Potatoes
Source: Int J Mol Sci. 2024 Jul 10;25(14):7584. doi: 10.3390/ijms25147584 (PMC11276909; doi:10.3390/ijms25147584)
Supplement: Supplementary file 1 [file ijms-25-07584-s001.zip › ijms-3067227-supplementary.pdf]

Table S1. The value of the L* parameter of juices from red and purple-fleshed potatoes with the addition of fruit concentrates from lemon and lime and vegatable concentrates from rhubarb in the amount of 1%, 2%, 3%.

Flesh color	Concentration	Lemon			Lime			Rhubarb		
		0h	1h	4h	0h	1h	4h	0h	1h	4h
purple	1%	0.22	0.23	0.29	0.23	0.20	0.29	0.17	0.16	0.24
	2%	0.16	0.05	0.09	0.06	0.05	0.12	0.07	0.18	0.24
	3%	0.06	0.07	0.06	0.14	0.10	0.21	0.06	0.14	0.21
red	1%	4.13	4.49	9.52	1.64	5.10	9.14	5.25	10.42	13.45
	2%	5.14	11.13	10.10	1.43	2.24	11.21	3.52	10.52	16.14
	3%	2.75	2.34	3.81	1.11	1.56	3.87	0.54	10.85	15.33

Table S2. Color of potato juices without additives (control sample) and with the addition of fruit and vegetable concentrates immediately after preparation, 1 hour and 4 hours after preparation.

		0h				1h				4h			
Flesh color	Variety	a*	b*	C	h°	a*	b*	C	h°	a*	b*	C	h°
purple	control sample	-0.05^{a}	0.07^{a}	0.14^{a}	190.25^{a}	0.03^{a}	0.05^{b}	0.17^{a}	143.28^{a}	0.14^{a}	-0.01^{b}	0.21^{a}	106.77^{a}
	lemon 1%	0.45^{b}	-0.19^{b}	0.53^{b}	273.56^{ab}	0.59^{c}	-0.08^{ab}	0.63^{c}	199.65^{a}	0.75^{b}	-0.07^{ab}	0.76^{c}	295.05^{b}
	lime 1%	0.49^{b}	-0.18^{b}	0.56^{b}	248.04^{ab}	0.48^{c}	-0.05^{ab}	0.52^{c}	176.62^{a}	0.99^{b}	-0.05^{ab}	0.96^{c}	238.21^{b}
	rhubarb 1%	0.42^{b}	-0.28^{b}	0.52^{b}	326.0^{b}	0.28^{b}	-0.14^{a}	0.36^{b}	218.90^{a}	0.39^{a}	-0.19^{a}	0.47^{b}	275.54^{b}
	LSD	0.17	0.15	0.16	101.42	0.16	0.17	0.16	144.25	0.25	0.15	0.22	129.73
red	control sample	4.48^{a}	1.45^{a}	4.71^{a}	17.83^{a}	2.33^{a}	0.69^{a}	2.43^{a}	16.39^{a}	1.75^{a}	0.52^{a}	1.83^{a}	16.79^{a}
	lemon 2%	17.23^{b}	6.11^{b}	18.28^{b}	19.49^{b}	18.78^{b}	7.56^{b}	20.26^{b}	20.90^{b}	25.08^{c}	15.94^{c}	29.95^{c}	27.41^{b}
	lime 2%	6.85^{a}	2.41^{a}	7.26^{b}	19.39^{b}	10.89^{a}	3.76^{a}	11.49^{b}	19.19^{b}	30.97^{b}	19.00^{b}	36.65^{b}	28.16^{b}
	rhubarb 2%	15.25^{b}	5.57^{a}	16.27^{b}	20.23^{b}	30.95^{b}	17.18^{a}	35.41^{b}	29.06^{b}	37.54^{d}	25.86^{d}	45.66^{d}	34.04^{c}
	LSD	3.2	1.29	3.43	1.22	3.2	1.29	3.43	1.22	3.65	1.76	4.02	1.81

Data are expressed as the mean. n=12. Results in the same column followed by different letters indicate significant differences according to Duncan's test at $p < 0.05$ between different flesh colors and variants. as determined by one-way ANOVA. LSD- last significant difference.

Table S3. Color of pigments in potato juices without additives (control sample) and with the addition of fruit and vegetable concentrates immediately after preparation, 1 hour and 4 hours after preparation.

		0h				1h				4h			
Flesh color	Variety	a*	b*	C	h°	a*	b*	C	h°	a*	b*	C	h°
purple	control sample	10.17^{d}	-10.78^{a}	14.82^{d}	313.3^{c}	9.88^{d}	-10.66^{a}	14.53^{d}	312.9^{c}	9.59^{d}	-10.29^{a}	14.07^{d}	313.0^{c}
	lemon 1%	6.31^{c}	-5.65^{b}	8.48^{c}	318.1^{d}	6.95^{c}	-5.70^{b}	9.00^{c}	320.6^{b}	6.40^{c}	-5.63^{b}	8.53^{c}	318.6^{d}
	lime 1%	2.55^{b}	-4.83^{c}	5.46^{b}	297.8^{b}	2.56^{b}	-4.67^{c}	5.32^{b}	298.8^{d}	2.26^{b}	-4.44^{c}	4.98^{b}	296.9^{b}
	rhubarb 1%	0.77^{a}	-3.72^{d}	3.80^{a}	281.6^{a}	0.64^{a}	-3.64^{d}	3.70^{a}	279.9^{a}	1.00^{a}	-4.05^{c}	4.18^{a}	283.8^{a}
	LSD	0.69	0.6	0.87	2.42	0.5	0.58	0.69	2.93	0.25	0.43	0.44	1.93
red	control sample	15.98^{a}	6.32^{a}	17.34^{a}	21.42^{c}	16.1^{a}	6.19^{a}	17.42^{a}	20.98^{c}	16.31^{a}	5.92^{a}	17.57^{a}	20.05^{c}
	lemon 2%	37.09^{c}	4.72^{a}	38.57^{c}	7.32^{a}	37.16^{c}	4.55^{a}	38.39^{c}	6.80^{a}	36.86^{c}	4.10^{a}	38.05^{c}	6.61^{a}
	lime 2%	38.70^{d}	9.14^{b}	39.89^{c}	13.52^{b}	38.20^{c}	8.55^{b}	39.27^{c}	12.96^{b}	38.01^{c}	8.36^{b}	39.05^{c}	12.74^{b}
	rhubarb 2%	34.92^{b}	6.24^{a}	35.58^{b}	9.98^{ab}	34.39^{b}	5.50^{a}	34.91^{b}	8.99^{ab}	34.28^{b}	5.41^{a}	34.8^{b}	8.82^{ab}
	LSD	1.43	1.97	1.6	4.27	1.79	1.86	1.83	4.36	1.76	1.94	1.79	4.7

Data are expressed as the mean. n=12. Results in the same column followed by different letters indicate significant differences according to Duncan's test at $p < 0.05$ between different flesh colors and variants. as determined by one-way ANOVA. LSD- last significant difference.

Table S4. pH value of juices without additives (control sample) and with the addition of fruit and vegetable concentrates.

Concentrate	Variety of potato juice	pH
control sample	Violet Queen	6.2
lemon 1%	Violet Queen	4.3
lime 1%	Violet Queen	4.3
rhubarb 1%	Violet Queen	4.7
control sample	Mulberry Beauty	6.2
lemon 2%	Mulberry Beauty	3.5
lime 2%	Mulberry Beauty	3.7
rhubarb 2%	Mulberry Beauty	4.2
control sample	Magenta Love	6.0
lemon 2%	Magenta Love	3.4
lime 2%	Magenta Love	3.4
rhubarb 2%	Magenta Love	3.3
